# XiaoEr LianHuaQinqGan alleviates viral pneumonia in mice infected by influenza A and respiratory syncytial viruses

**DOI:** 10.1080/13880209.2022.2147961

**Published:** 2022-11-29

**Authors:** Wenyan Li, Tongtong Li, Chi Zhao, Tao Song, Yao Mi, Zhang Chuangfeng, Yunlong Hou, Zhenhua Jia

**Affiliations:** a Hebei Yiling Hospital, Hebei University of Chinese Medicine, Shijiazhuang, Hebei, China; b Shijiazhuang Yiling Pharmaceutical Co., Ltd, Shijiazhuang, Hebei, China; c College of Integrated Traditional Chinese and Western Medicine, Hebei University of Chinese Medicine, Shijiazhuang, Hebei, China; d Hebei Medical University, Shijiazhuang, Hebei, China; e National Key Laboratory of Collateral Disease Research and Innovative Chinese Medicine, Shijiazhuang, Hebei, China; f Shijiazhuang Compound Traditional Chinese Medicine Technology Innovation Center, Shijiazhuang, Hebei, China; g Hebei Yiling Hospital, Shijiazhuang, Hebei, China

**Keywords:** Xiaoer lianhuaqinggan(XELH), antiviral effects, anti-inflammatory effects, immunoregulatory effects

## Abstract

**Context:**

Xiaoer lianhuaqinqgan (XELH), developed based on Lianhua Qingwen (LHQW) prescription, contains 13 traditional Chinese medicines. It has completed the investigational new drug application to treat respiratory viral infections in children in China.

**Objective:**

This study demonstrates the pharmacological effects of XELH against viral pneumonia.

**Materials and methods:**

The antiviral and anti-inflammatory effects of XELH were investigated *in vitro* using H3N2-infected A549 and LPS-stimulated RAW264.7 cells and *in vivo* using BALB/c mice models of influenza A virus (H3N2) and respiratory syncytial virus (RSV)-infection. Mice were divided into 7 groups (*n* = 20): Control, Model, LHQW (0.5 g/kg), XELH-low (2 g/kg), XELH-medium (4 g/kg), XELH-high (8 g/kg), and positive drug (20 mg/kg oseltamivir or 60 mg/kg ribavirin) groups. The anti-inflammatory effects of XELH were tested in a rat model of LPS-induced fever and a mouse model of xylene-induced ear edoema.

**Results:**

*In vitro,* XELH inhibited the pro-inflammatory cytokines and replication of H1N1, H3N2, H1N1, FluB, H9N2, H6N2, H7N3, RSV, and HCoV-229E viruses, with (IC_50_ 47.4, 114, 79, 250, 99.2, 170, 79, 62.5, and 93 μg/mL, respectively). *In vivo*, XELH reduced weight loss and lung index, inhibited viral replication and macrophage M1 polarization, ameliorated lung damage, decreased inflammatory cell infiltration and pro-inflammatory cytokines expression in lung tissues, and increased the CD4^+^/CD8^+^ ratio. XELH inhibited LPS-induced fever in rats and xylene-induced ear edoema in mice.

**Conclusion:**

XELH efficacy partially depends on integrated immunoregulatory effects. XELH is a promising therapeutic option against childhood respiratory viral infections.

## Introduction

Infants and young children are particularly susceptible to respiratory infections, which are highly associated with hospitalization and mortality and impose a large burden on social and medical resources (Lambert et al. 2008; Tregoning and Schwarze 2010). Among all pathogenic factors, viruses are considered the main pathogens. The most frequently detected viruses responsible for acute respiratory infections are respiratory syncytial virus (RSV), rhinovirus, and influenza virus (Passioti et al. 2014). As the coronavirus disease 2019 (COVID-19) spreads, respiratory infections by newly emerging viruses can become a pandemic worldwide and more deadly than the viruses mentioned above (Passioti et al. 2014; Unger and Bogaert 2017). Unfortunately, because of the lack of effective preventative or curative medicines for viral infections, mortality and morbidity due to respiratory infections are still greater than other diseases among children (World Health Organization [WHO] 2005; Meissner 2016). Although scientific advances have been made in understanding the aetiological attributes and epidemiological characteristics of respiratory virus infections, it is challenging to solve the urgent problem of the shortage of antiviral medicines.

About 870,000 children under the age of 5 years are hospitalized worldwide each year because of influenza alone (Lafond et al. 2016). Although respiratory virus infections can occur at all ages, the rate of viral infections is consistently high in children owing to their immature immunity and immune activity responses and the characteristics of the viruses themselves (WHO 2005; Guerche-Séblain et al. 2019). For example, for RSV and influenza virus, vaccines are minimally effective in children than in adults because of the differences in their immune activities and responses and the characteristics of the viruses (Adkins et al. 2004; Siegrist 2007). RSV has a single-stranded RNA that is error-prone and has no proofreading mechanism, which results in the rapid generation of single nucleotide polymorphisms (SNPs) and other mutations (Piedimonte 2015). In addition, the rapid evolution of influenza viruses and the annual emergence of novel strains have led to changes in virus virulence and the invalidation of antiviral vaccines (Krammer et al. 2018). Moreover, virus mutagenicity may also defunct antiviral agents. For instance, only a single point mutation in the neuraminidase gene of the N1 influenza virus confers resistance to oseltamivir (Hurt et al. 2012). In addition to immature immunity, the lack of prior exposure in children leads to the failure of the innate immune system to detect viruses and reduces the immune responses compared to that in adults (Ofer 2007).

Traditional Chinese medicine (TCM) has long been used to treat various respiratory virus-infected diseases (Yang et al. 2020). TCM, with multiple components and targets, exerts broad-spectrum antiviral activities and immunomodulatory effects. It plays an important role in preventing disease development and shortening the treatment cycle (Poon et al. 2006; Jun et al. 2014). For example, Lianhuaqingwen (LHQW), a TCM formula used to treat influenza, has broad-spectrum antiviral and immunomodulatory effects against various influenza viruses (Ding et al. 2017). It has been reported that the antiviral effect of LHQW is mainly exerted through its NF-kB inhibiting activity to restrain viral ribonucleoprotein (RNP) export and subsequent viral propagation. Moreover, LHQW blocks proapoptotic communication between macrophages and alveolar epithelial cells by inhibiting endoplasmic reticulum (ER) stress and tumour necrosis factor-related apoptosis-inducing ligand secretion in an acute lung injury model (Li, Ran, et al. 2020). In severe acute respiratory syndrome coronavirus 2 (SARS-Cov-2) infection, LHQW was shown to significantly relieve cardinal symptoms, reduce the course of COVID-19, and regulate the inflammatory response as well as immune function (Li, Hou, et al. 2020; Li, Zhang, et al. 2020).

XiaoEr LianHuaQinqGan (XELH) was developed by Shijiazhuang Yiling Pharmaceutical Co., Ltd. (Shijiazhuang, China) based on the prescription of LianhuaQingwen (LHQW), which has been proven to inhibit many influenza strains *in vitro*. Presently, the investigational new drug application (IND) for XELH has been approved, and its clinical trials are under process. The XELH prescription includes *Forsythia suspensa* (Thunb.) Vahl (Oleaceae), *Isatis indigotica* Fort. (Brassicaceae), *Lonicera hypoglauca* Miq. (Caprifoliaceae), *Pogostemon cablin* Benth. (Lamiaceae), *Mentha haplocalyx* Briq. (Lamiaceae), *Rhodiola rosea* L. (Crassulaceae), *Ephedra sinica* Stapf (Ephedraceae), *Rheum palmatum* L. (Polygonaceae), *Glycyrrhiza uralensis* Fisch. (Fabaceae), Gypsum fibrosum, and Semen Armeniacae Amarum [the dried, ripe seeds of *Prunus armeniaca* L. (Rosaceae)]. Preliminary pharmacodynamic results showed that XELH exerted broad-spectrum antiviral and anti-inflammatory effects *in vitro* and *in vivo* (data used for IND). However, its safety for application in children has not been explored. In the present study, we evaluated the antiviral effect of XELH and investigated the mechanism of its protective action against viral infection *in vitro* and *in vivo*, providing evidence for further clinical applications.

## Materials and methods

### Reagent preparation

XELH powder (lot no. 191101) was obtained from Shijiazhuang Yiling Pharmaceutical Co., Ltd. The powder was dissolved in dimethyl sulfoxide (DMSO) to a concentration of 61.25 mg/mL by ultrasonic heating and stored at −20 °C. Ribavirin was obtained from MedChemExpress (lot no. #11970), and Oseltamivir was obtained from Roche Registration (lot no. M1059).

**Figure 1.**
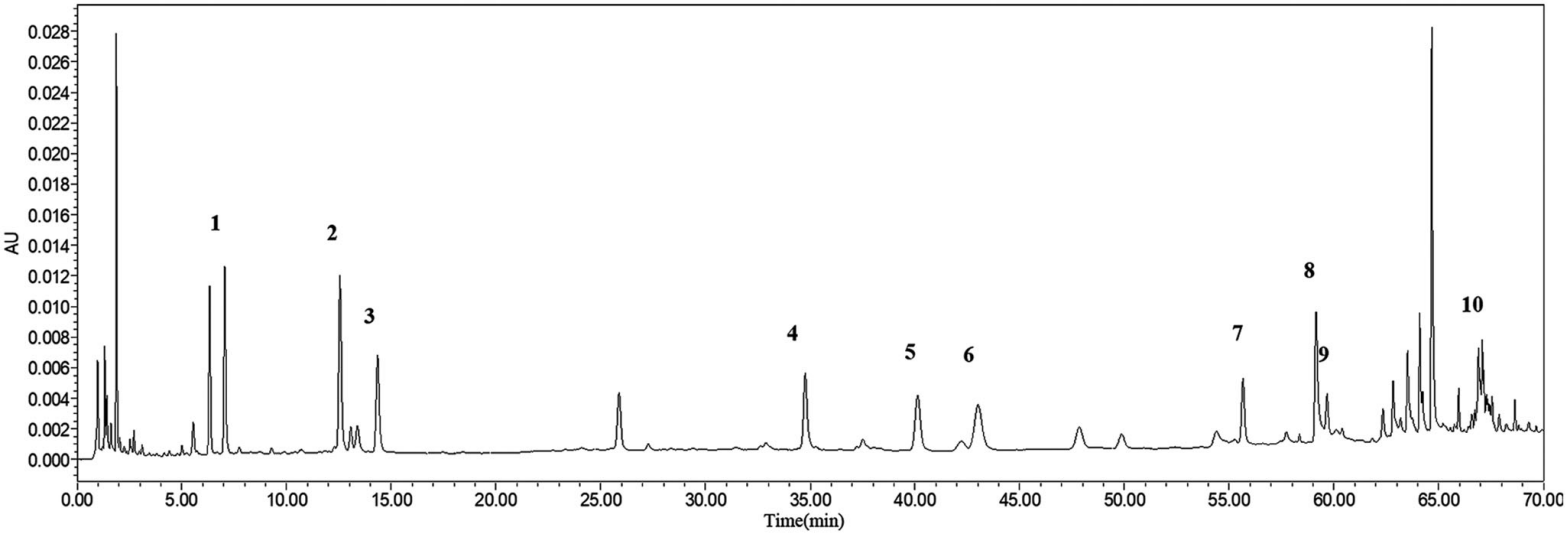
The ultra-high performance liquid chromatography fingerprints of XiaoEr LianHuaQinqGan (XELH) showed 10 chromatogram peaks: (**1**) Neochlorogenic acid, (**2**) Chlorogenic acid, (**3**) Cryptochlorogenic acid, (**4**) Isoforsythiaside, (**5**) Forsythoside A, (**6**) Verbascoside, (**7**) 4,5-Di-*O*-caffeoylquinic acid, (**8**) Physcion, (**9**) Phillyrin, (**10**) Glycyrrhizic acid.

The ultra-high performance liquid chromatography (UPLC) analysis was performed to test the main components of XELH. We investigated the main components of the prescription in the literature (Jia et al. 2015) and purchased the reference samples from Shanghai Shidande Standard Technical Service Co., Ltd. (Shanghai, China). These components included neochlorogenic acid (ST06230120), chlorogenic acid (RS02151120), cryptochlorogenic acid (ST07850120), isoforsythiaside (ST19190120), forsythoside A (RS00891020), verbascoside (RS00301020), 3,4-dicaffeoylquinic acid (RS06601020), physcion (RS01411020), phillyrin (RS02071020), and glycyrrhizic acid (RS00661020). Forsythoside A was used as quality control to ensure batch-to-batch stability considering its stability. The reference samples and XELH were analysed according to the standard characteristic fingerprint of LHQW capsules (Gao et al. 2020). Briefly, in a conical flask, 0.25 g XELH powder was dissolved in 25 mL 50% methanol by ultrasonic for 30 min. After cooling, the lost volume was supplemented with 50% methanol and shaken. The solution was then filtered using a 0.22 μm microporous filter membrane, and the filtrate was used for the UPLC analysis. The separation was performed on a Waters H-Class system (Waters Corporation, Milford, MA, USA) fitted with an ACQUITY UPLC® HSS T3 column (1.8 μm, 2.1 × 100 mm; Waters Corporation) using methanol, 0.1% phosphoric acid, and acetonitrile as mobile phase. The flow rate was 0.3 mL/min, the column temperature was 30 °C, the injection volume was 2 μL, and the detection wavelength at 239 nm. The UPLC analysis of XELH showed 10 chromatogram peaks (Figure 1): neochlorogenic acid (**1**), chlorogenic acid (**2**), cryptochlorogenic acid (**3**), isoforsythiaside (**4**), forsythoside A (**5**), verbascoside (**6**), 4,5-Di-*O*-caffeoylquinic acid (**7**), physcion (**8**), phillyrin (**9**), and glycyrrhizic acid (**10**). The 10 chromatographic peaks were confirmed by comparison with the reference samples.

### Virus

A/PR/8/34 (H1N1), A/GZ/GIRD/07/2009 (H1N1), A/California/2/2014 (H3N2), B/Lee/1940 (FluB), A/chicken/Guangdong/1996/(H9N2, H6N2, H7N3), HCoV-229E, and respiratory syncytial virus (RSV) were obtained from the American Type Culture Collection (ATCC; Manassas; VA, USA). The viruses were propagated in host cells, and the viral titres were determined using the Reed-Muench method with a 50% tissue culture infective dose (TCID50) (Yu et al. 2018).

### Cytotoxicity assay

The methyl thiazolyl tetrazolium (MTT) assay was performed to test the cytotoxic effects of XELH *in vitro*. The cytotoxic effects of XELH on Madin-Darby canine kidney (MDCK), Hep-2, Vero, Medical Research Council cell strain 5 (MRC-5), and Huh-7 cells were tested. These cells were inoculated in 96-well plates and washed with phosphate-buffered saline (PBS) when they grew to a monolayer. They were then divided into different groups (*n* = 4 wells per group) and incubated for 48 h with the different concentrations of XELH (5, 2.5, 1.25, 0.625, 0.3125, and 0.15625 mg/mL). Afterward, the cells were stained with 1 mg/mL MTT solution for 4 h, and 100 µL DMSO was added to each well after removing the supernatant. Finally, absorbance was measured at 490 nm using a full-wavelength scanner microplate reader (Infinite M200 Pro, Tecan, Switzerland).

### Cytopathic effect (CPE) inhibition assay

The cytopathic effect (CPE) inhibition assay was performed to detect the antiviral activity of XELH. Briefly, the cells were incubated in 96-well plates for 24 h, after which 100 TCID_50_ virus diluent was added and incubated for 2 h. Subsequently, different concentrations of XELH (2500, 1250, 625, 312.5, 156.25, 78.125, 39.0625, and 19.5325 μg/mL) were added for 48 h (*n* = 4 wells per group). The Reed-Muench method was used to calculate the half-inhibitory concentration (IC_50_).

### Cell culture, RNA extraction, and reverse transcription PCR (RT-PCR) analysis

The reverse transcription PCR (RT-PCR) analysis was performed to detect the anti-inflammatory effect of XELH *in vitro*. A549 cells were incubated in 6-well plates for 48 h, washed twice with PBS, and then exposed to H3N2 at 0.02 a multiplicity of infection (MOI) for 2 h at 37 °C. The supernatant was removed and replaced with the indicated concentration (10, 100, 1000 μg/mL) of XELH for 24 h. The RAW 264.7 cells were incubated in 6-well plates for 48 h, washed with PBS, and incubated with a serum-free medium for 24 h. The indicated concentrations of lipopolysaccharide (LPS) and different concentrations of XELH were added for 24 h. Total RNA was extracted from each group of cells (*n* = 3 wells per group) using Trizol reagent (Invitrogen, USA) and reverse-transcribed into cDNA using a GoScriptTM reverse-transcription system (Promega, USA) according to the manufacturer’s protocols. qRT-PCR was performed on an ABI 7500 system using the following cycling conditions: Pre-denaturation 95 °C, 30 s; denaturation 95 °C, 5 s; annealing and elongation 60 °C, 40 s; a total of 40 cycles. The primers (Table 1) were synthesized from Wanlei Biotechnology Co. Ltd. (Shenyang, China). *GAPDH* was used as the internal reference, and the relative mRNA expression was calculated using the 2^−ΔΔCt^ method.

**Table 1.** List of Cytokine primer sequences.

Gene	Primer	Sequence (5′–3′)
*CCL5*	Fwd	CAGCAGTCGTCTTTGTCACC
	Rev	GTTGATGTACTCCCGAACCC
*GAPDH*	Fwd	GAAGGTGAAGGTCGGAGTC
	Rev	GAAGATGGTGATGGGATTTC
*IL-6*	Fwd	CGGGAACGAAAGAGAAGCTCTA
	Rev	CGCTTGTGGAGAAGGAGTTCA
*TNF-α*	Fwd	AACATCCAACCTTCCCAAACG
	Rev	GACCCTAAGCCCCCAATTCTC
*IP-10*	Fwd	GAAATTATTCCTGCAAGCCAATTT
	Rev	TCACCCTTCTTTTTCAT-TGTAGCA
*MCP*	Fwd	CAAGCAGAAGTGGGTTCAGGAT
	Rev	AGTGAGTGTTCAAGTCTTCGGAGTT
*MIP-1β*	Fwd	AAAACCTCTTTGCCACCAATACC
	Rev	GAGAGCAGAAGGCAGCTACTAG
*RIG-I*	Fwd	GATGCTCTGGATTACTTG
	Rev	GTGGTACTCTTCTTGTAAG
*IL-1β*	Fwd	GCACGATGCACCTGTACGAT
	Rev	AGACATCACCAAGCTTTTTTGCT
*CXCL1*	Fwd	GGGTACATTATGGAGGCTTTCTCA
	Rev	GAGGACGCTGTCTTTGCATAGG

### Immunofluorescence assay

The immunofluorescence assay was performed to test the inhibitory effect of XELH on viral nucleoprotein (NP) expression *in vitro*. MDCK cells were incubated in 8-well plates, washed with PBS, and then exposed to 1 MOI H3N2 for 2 h at 4 °C. The cells were washed with PBS and a medium containing TPCK treated-trypsin (Sigma-Aldrich, Germany; cat. no. 232-650-8). XELH (1 mg/mL) containing TPCK was added 0, 2, 4, 6, and 8 h after infection. After 10 h, the slides were washed twice with PBS. Next, 4% paraformaldehyde was added for 20 min at room temperature (25 °C). The sections were then washed three times with PBS, incubated with 0.5% Triton-X100 for 10 min at room temperature, and washed three times with PBS. Next, 5% goat serum was added for 20 min at room temperature. The sections were incubated with anti-influenza A virus NP antibody (Abcam; cat. no. ab20343) at 4 °C overnight, followed by incubation with a fluorescein-conjugated affinipure goat anti-mouse IgG antibody (Proteintech; cat. no. SA00003-1) for 1 h at room temperature. Finally, the cells were labelled with 4′,6-diamidino-2-phenylindole (DAPI) glycerin-staining solution and observed under a fluorescence microscope (Nikon 942101).

### Time-course analysis for XELH

The time-course analysis was performed to test the inhibitory effects of XELH on virus replication *in vitro*. Briefly, MDCK cells were incubated in 12-well plates, washed twice with PBS, and 1 MOI H3N2 was added for 2 h at 4 °C. Cells were washed twice with cold PBS, the medium was added, and the cells were incubated in a CO_2_ incubator at 37 °C. XELH (1 mg/mL) was added 0, 2, 4, 6, and 8 h after infection. After 10 h, the 12-well plates were frozen and thawed three times, and the samples were collected at each time point for TCID_50_.

### Establishment of mouse models of H3N2- and RSV- infection and treatment

To evaluate the antiviral effect of XELH *in vivo*, we established the H3N2- and RSV- infected mouse models. Four-week-old BALB/c mice weighing 14-21 g (*n* = 364) were purchased from Hunan SJA Laboratory Animal Co. Ltd. (Hunan, China) [Production certificate No.: SCXK (Xiang) 2019-0004].

Of the 364 mice, 140 [male (*n* = 70); female (*n* = 70); weighing 15.6-20.0 g] were used to test the efficacy of XELH against H3N2 infection. The mice were divided into 7 groups (*n* = 20), Normal, Model, Oseltamivir (positive drug), LHQW, XELH-Low, XELH-Medium, and XELH-High, based on the drugs with which they were treated. Mice in Oseltamivir, LHQW, XELH-Low, XELH-Medium, and XELH-High groups (treatment groups) were treated with 20 mg/kg oseltamivir (Roche Registration; 204255-11-8), 0.5 g/kg LHQW, 2, 4, and 8 g/kg XELH, respectively, once a day, for 7 consecutive days. Mice in Normal and Model groups were administered with distilled water. On day 4 (D4) of the experiment, the mice in the treatment and Model groups were slightly anaesthetized with ether, and 80 μL H3N2 virus stock solution [the hemagglutination assay (HA) titre of H3N2 was 6log_2_] was dropped through their nose. The same volume of blank medium was dropped through the nose of the mice in the Normal group. The detection indices were estimated at 2 h after the last administration of respective medications on D5 (24 h after the virus infection) and D7 (72 h after the virus infection).

The remaining 224 mice [male (*n* = 112); female (*n* = 112); weighing 14.4-20.3 g] were used for testing the efficacy of XELH against RSV infection. As described above, based on the drugs they were treated with, the mice were divided into seven groups (*n* = 32), namely Normal, Model, Ribavirin (positive drug), LHQW, XELH-Low, XELH-Medium, and XELH-High. Mice in Ribavirin group were treated with 60 mg/kg ribavirin (Shandong Qidu Pharmaceutical Co., Ltd., Shandong, China), wherein those in LHQW, XELH-Low, XELH-Medium, and XELH-High groups were treated with respective medications as described above. Mice in Normal and Model groups were administered with distilled water. On D4, the mice in the treatment (Ribavirin, LHQW, XELH-Low, XELH-Medium, and XELH-High) and Model groups were slightly anaesthetized with ether, and 80 μL (virus concentration 100 TCID_50_) RSV stock solution was dropped through the nose. Mice in Normal group were treated with the same volume of blank medium administered through nasal drops. The detection indices were estimated on D5 and D7, as described above.

The experimental procedures and animal welfare were in accordance with the Ethics Review Committee for Animal Experimentation of Drug Safety Evaluation and Research Centre of Hunan Province (approval number: IACUC-2020(2)027). The experimental protocols were approved by the Provincial Drug Safety Evaluation Animal Experiment Ethics Review Committee and Hunan Provincial Research Centre (protocol number: HNSE2020(2)027).

### HA titre determination of H3N2 in vivo

Mice (*n* = 10) in each group were sacrificed by cervical dislocation on D5 and D7. Both lungs were harvested aseptically, and individual lung (right and left lungs were processed separately) suspensions were prepared. The harvested lungs were placed in a homogenizer, and sodium chloride injection (0.9%) was added according to the lung mass (g) in a ratio of 9:1. Then, the lungs were homogenized to a suspension by manual grinding. The hemagglutination inhibition test was used to detect the hemagglutination inhibition titre of lung suspension (taking the maximum dilution multiple of complete inhibition of erythrocyte aggregation as the hemagglutination inhibition titre), and log_2_ (maximum dilution multiple) represented the virus titre in the lung.

### Viral load determination of H3N2 and RSV in vivo

RT-PCR analysis was performed to detect the antiviral activity of XELH *in vivo*. The H3N2 and RSV viral loads in lung tissues of mice (*n* = 10 in each group) were measured using RT-PCR. Total RNA was extracted using a Fast Pure Cell/Tissue Total RNA Isolation Kit (Vazyme, Nanjing, China). The reverse transcription was performed using the High Capacity cDNA Reverse Transcription kit (Thermo Fisher Scientific, MA, USA; cat. no. 4368813). qRT-PCR was performed on an ABI 7500 system using the following cycling conditions: Pre-denaturation 95 °C, 30 s; denaturation 95 °C, 5 s; annealing and elongation 55 °C, 40 s; a total of 40 cycles. The primers (Table 2) were synthesized from Liuhe Huada Gene Technology Co., Ltd. (Beijing, China).

**Table 2.** List of H3N2 and RSV primer sequences.

Gene	Primer	Sequence (5′–3′)
H3N2-*NP*	Fwd	GACCCTTTCAAACTACTTC
	Rev	GAAGCAATTTGTACTCCTC
RSV-*F*	Fwd	AATCGAGCCAGAAGAGAACTA
	Rev	GCCTTGTTTGTGGATAGTAGAG

### Enzyme-linked immunoassay (ELISA) in lung tissues of H3N2 and RSV-infected mice

The enzyme-linked immunoassay (ELISA) analysis was performed to detect the anti-inflammatory effect of XELH *in vivo.* Homogenate of right lung tissues of H3N2- and RSV-infected mice (*n* = 10 in each group) were prepared as described in the ‘*HA titre determination of H3N2 in vivo’* section. The levels of IL-1β (cat. no. MM-0040M1), TNF-α (cat. no. MM-0132M1), IFN-γ (cat. no. MM-0182M1), and IL-10 (cat. no. MM-0176M1) in lung tissues were detected using corresponding ELISA Kits (Meimian, Jiangsu, China).

### Histopathological analysis

The histopathological analysis was performed to detect the ameliorating effects of XELH on the lung pathology of virus-infected mice. Left lung tissues of H3N2- and RSV-infected mice (*n* = 10 in each group) were harvested after euthanizing by cervical dislocation on D5 and D7. Then, the tissue samples were perfused with 10% formalin and embedded in paraffin. Afterward, the samples were cut into 3-5 µm thick sections, stained with haematoxylin and eosin (H&E), and the pathological changes were observed using a microscope (NanoZoomer, Hamamatsu Photonics, Japan).

### Immunofluorescence staining of lung tissue sections for CD4^+^, CD8^+^

The immunofluorescence staining analysis was performed to detect the ability of XELH to improve the imbalance of CD4^+^ and CD8^+^ lymphocytes in the lung tissue of RSV-infected mice. Lung tissues (*n* = 6 in each group) were harvested as described in the previous section (*HA titre determination of H3N2 in vivo*), frozen in optimal cutting temperature (OCT) compound (-20 °C). The frozen tissue block was sliced into 10 μm sections using a cryostat microtome (Leica CM1950) at -20 °C and attached to an ITO-coated glass slide. The slide was washed briefly in PBS, Immunol staining blocking buffer (Beyotime Biotechnology; Shanghai; cat. no. P0102) was added for 1 h, and incubated overnight with the CD4 monoclonal antibody (Thermo Fisher; cat. no. 42-0042-82), CD8 alpha monoclonal antibody (Thermo Fisher; cat. no. MA5-18153) at 4 °C. Afterward, the slides were washed with PBS, labelled using DAPI glycerin-staining solution, and observed under confocal microscopy (Zeiss LSM710).

### Classification of macrophage cells using flow cytometry

The flow cytometry analysis was performed to assess the effect of XELH on macrophage M1 polarization inhibition. A homogenate of the whole lung was prepared as described previously (*HA titre determination of H3N2 in vivo*), followed by cell counting (*n* = 6 in each group). The cell density was adjusted, and the homogenate of the whole lung was incubated with F4/80 monoclonal antibody (BM8), PE (Thermo Fisher; cat. no. MF48004-3), and CD206 (MMR) antibody APC (Thermo Fisher; cat. no. 17-2069-42) according to the manufacturers’ instructions. F4/80-PE and CD206-APC expression levels were assessed using flow cytometry (BD FACSAria™ III, BD Biosciences).

### Determination of lung index in H3N2- and RSV-infected mice

Lung tissues were collected by euthanizing the mice of each group on D5 and D7 by cervical dislocation and weighed. Lung index was calculated using the following formula: Lung index = [lung wet weight (mg)/body weight (g)].

### Establishment of a rat model of LPS-induced fever and treatment

A rat model of LPS-induced fever was established to test the anti-inflammatory effect of XEHL *in vivo*. A total of 90 male SD rats (4 weeks old) were obtained from Beijing Vital River Laboratory Animal Technology Co., Ltd. (Beijing, China) [Production certificate no. SCXK (Jing) 2016-0006]. The basal temperature was measured at 0 min, and the rats were intraperitoneally injected with 20 μg/kg LPS (Sigma-Aldrich). After 1 h, the temperature was checked, and the rats were treated with indicated medications. The rats were divided into five groups (*n* = 18) based on their medication: Model (no treatment), Ibuprofen (26.64 mg/kg ibuprofen), XELH-High (6.40 g/kg XELH), XELH-Medium (3.20 g/kg XELH), and XELH-Low (1.60 g/kg XELH). The anal temperature was measured for 5 h at 1 h intervals (Tx), and the temperature difference was calculated as ΔT = Tx − T0.

### Establishment of a mouse model of xylene-induced ear edoema and treatment

A mouse model of xylene-induced ear edoema was also established to test the anti-inflammatory effect of XEHL *in vivo*. A total of 75 male KM mice (weighing 18-22 g, 4 weeks old) were obtained from Beijing Vital River Laboratory Animal Technology Co., Ltd. [Beijing, China; production certificate no. SCXK (Jing) 2016-0011]. Mice were divided into five groups (*n* = 15): Model, Aspirin (0.50 g/kg aspirin), XELH-High group (8.00 g/kg XELH), XELH-Medium (4.00 g/kg XELH) and XELH-Low (2.00 g/kg XELH) groups. Mice in Aspirin, XELH-High, XELH-Medium, and XELH-Low (treatment) groups were administered with 0.50 g/kg aspirin, 8.00, 4.00, and 2.00 g/kg XELH, respectively, continuously for 5 days. Mice in Model group were administered distilled water. After 30 min of the last administration of the drugs, xylene (Tianjin Damao Chemical Reagent Co., Ltd., Tianjin, China.) was smeared on the middle of the right ear (30 μL). After 40 min, the mice were euthanized by cervical dislocation, and their ears were harvested. The degree and rate of inhibition of swelling of the ears were calculated as follows:

Degree of swelling=The weight of the right ear−The weight of the left ear.

### Statistical analyses

The data are presented as mean ± standard deviation (SD). All analyses were performed using SPSS20.0 statistical software. The Student’s *t*-test was used to compare the data of two groups, and analysis of variance (ANOVA) was used to compare the data of multiple groups. Differences were compared using the least significant difference (LSD), Kruskal–Wallis, or Dunnett’s tests. The significance threshold was α = 0.05, and differences with *p* ≤ 0.05 were considered significant.

## Results

### Cytotoxicity and antiviral activity of XELH in vitro

**Figure 2.**
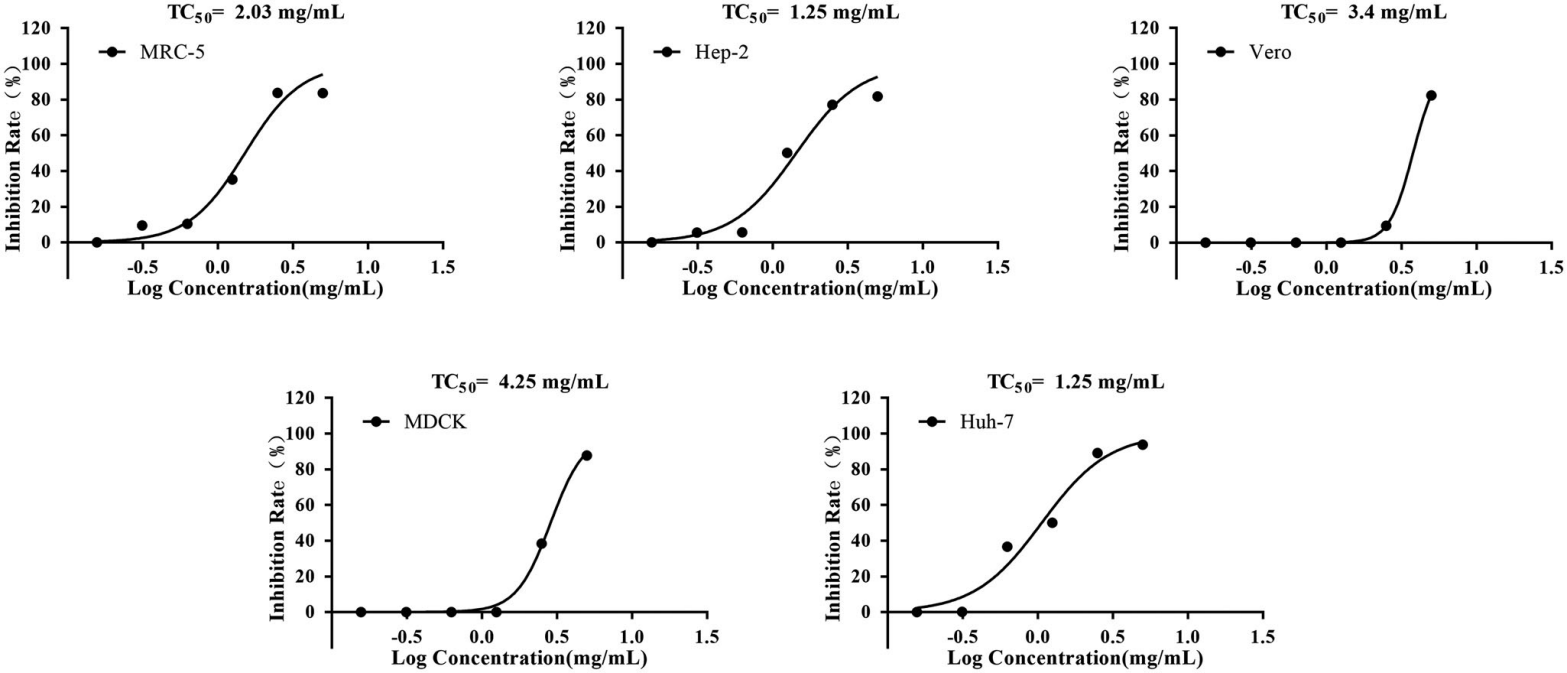
Cytotoxicity of XELH on the viability of MRC-5, Hep-2, Vero, MDCK, and Huh-7 cells assessed using methyl thiazolyl tetrazolium (MTT) assay. The cells were divided into different groups (*n* = 4 wells per group) and treated with different concentrations of XELH (5, 2.5, 1.25, 0.625, 0.3125, and 0.15625 mg/mL), incubated for 48 h, and MTT (1 mg/mL) was added to each well for 4 h. The TC_50_ values of XELH in MRC-5, Hep-2, Vero, MDCK, and Huh-7 cells are 2.03, 1.25, 3.4, 4.25, and 1.25 mg/mL, respectively.

The cytotoxicity of XELH on MRC-5, Hep-2, Huh-7, MDCK, and Vero cell viability was assessed using an MTT assay. The TC_50_ values of XELH in MRC-5, Hep-2, Huh-7, MDCK, and Vero cells were 2.03, 1.25, 1.25, 4.25, and 3.4 mg/mL, respectively (Figure 2).

CPE assay demonstrated that XELH inhibited the replication of A/PR/8/34 (H1N1), A/California/2/2014 (H3N2), A/GZ/GIRD/07/2009 (H1N1), B/Lee/1940 (FluB), A/Chicken/Guangdong/1996 (H9N2), A/Chicken/Guangdong/1996 (H6N2), A/Chicken/Guangdong/1996 (H7N3), RSV and HCoV-229E viruses with IC_50_ values of 47.4, 114, 79, 250, 99.2, 170, 79, 62.5, and 93 μg/mL, respectively. The selectivity index (SI; the ratio of the antiviral activity and cytotoxicity***)*** were 89.66, 37.28, 53.79, 17, 42.84, 25, 53.79, 16, and 13.44, respectively (Table 3). These results suggest that XELH has a broad-spectrum antiviral effect *in vitro*.

**Table 3.** Antiviral effect of XiaoEr LianHuaQinqGan.

Virus type	XELH		
	TC50 (μg/ml)	IC50 (μg/mL)	SI
H1N1	4250	47.4	89.66
H3N2	4250	114	37.28
H1N1	4250	79	53.79
FluB	4250	250	17
H9N2	4250	99.2	42.84
H6N2	4250	170	25
H7N3	4250	79	53.79
RSV	1250	62.5	16
hCoV-229E	1250	93	13.44

### Anti-inflammatory effect of XELH in vitro

#### XELH inhibits the expression of cytokine and chemokine in vitro

**Figure 3.**
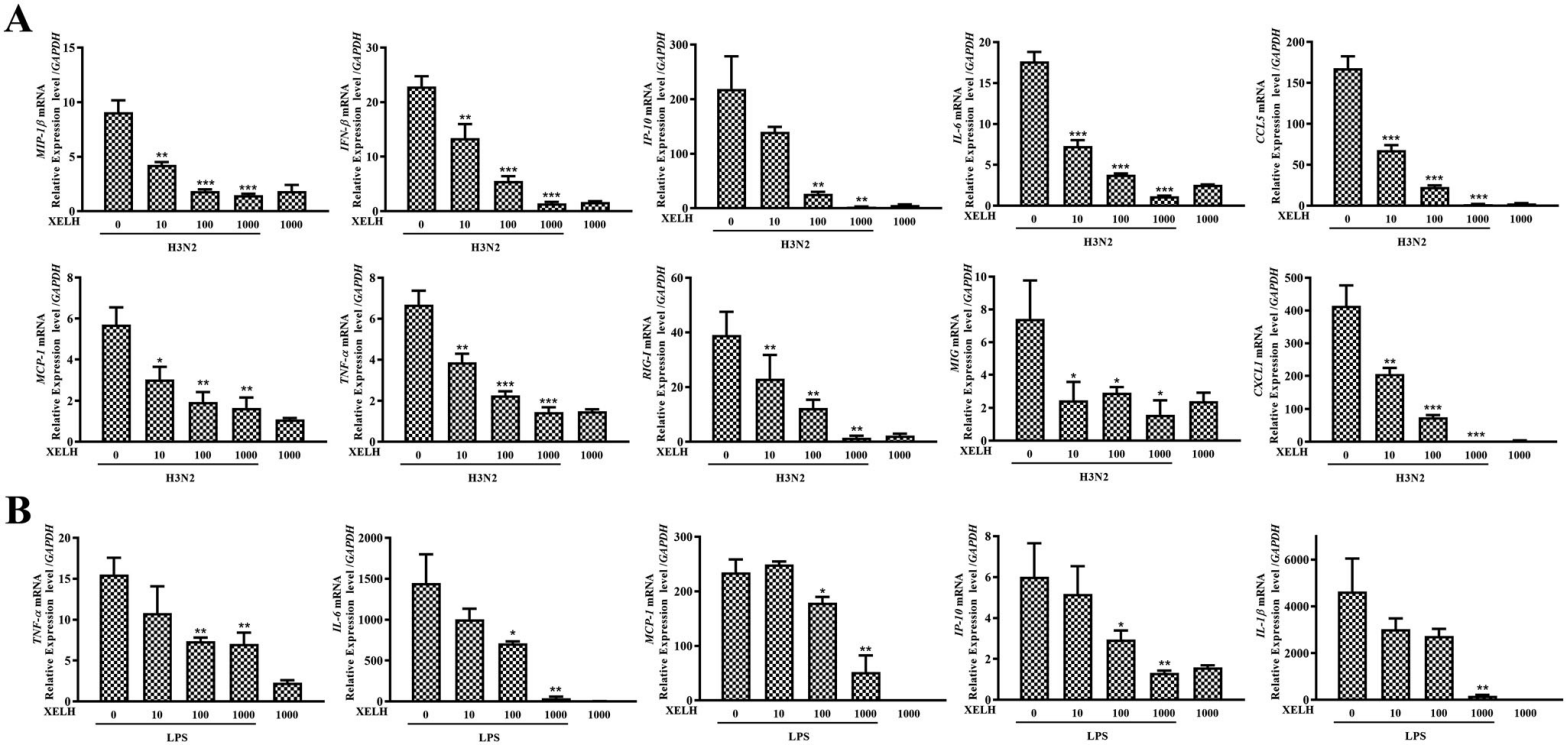
Anti-inflammatory effect of XELH *in vitro*. (A) Effects of XELH treatment (10, 100, 1000 μg/mL) on the mRNA expression of inflammatory mediators in H3N2-infected A549 cells. *p ≤ 0.05, **p ≤ 0.01, ***p ≤ 0.001, XELH vs. Model group (XELH 0 μg/mL). (B) Effect of XELH treatment (10, 100, 1000 μg/mL) on mRNA expression levels of inflammatory mediators in RAW 264.7 cells stimulated by lipopolysaccharide (LPS). Data are presented as mean ± standard deviation (SD) (*n* = 3 wells per group). *p ≤ 0.05, **p ≤ 0.01, ***p ≤ 0.001, XELH vs. Model group (XELH 0 μg/mL).

The mRNA expression levels of cytokines and chemokines induced by H3N2 and LPS were measured to assess the anti-inflammatory effect of XELH. As shown in Figure 3A, XELH significantly inhibited the elevated expression of *MIP-1β, IFN-β, IP-10, IL-6, CCL5, MCP-1, TNF-α, RIG-I, MIG*, and *CXCL1* induced by H3N2 infection in a dose-dependent manner. As shown in Figure 3B, XELH significantly inhibited LPS-induced elevated expression levels of *TNF-α, IL-6, MCP-1, IP-10*, and *IL-1β* stimulated by LPS in a dose-dependent manner.

#### XELH inhibits H3N2 virus replication in the early stage

**Figure 4.**
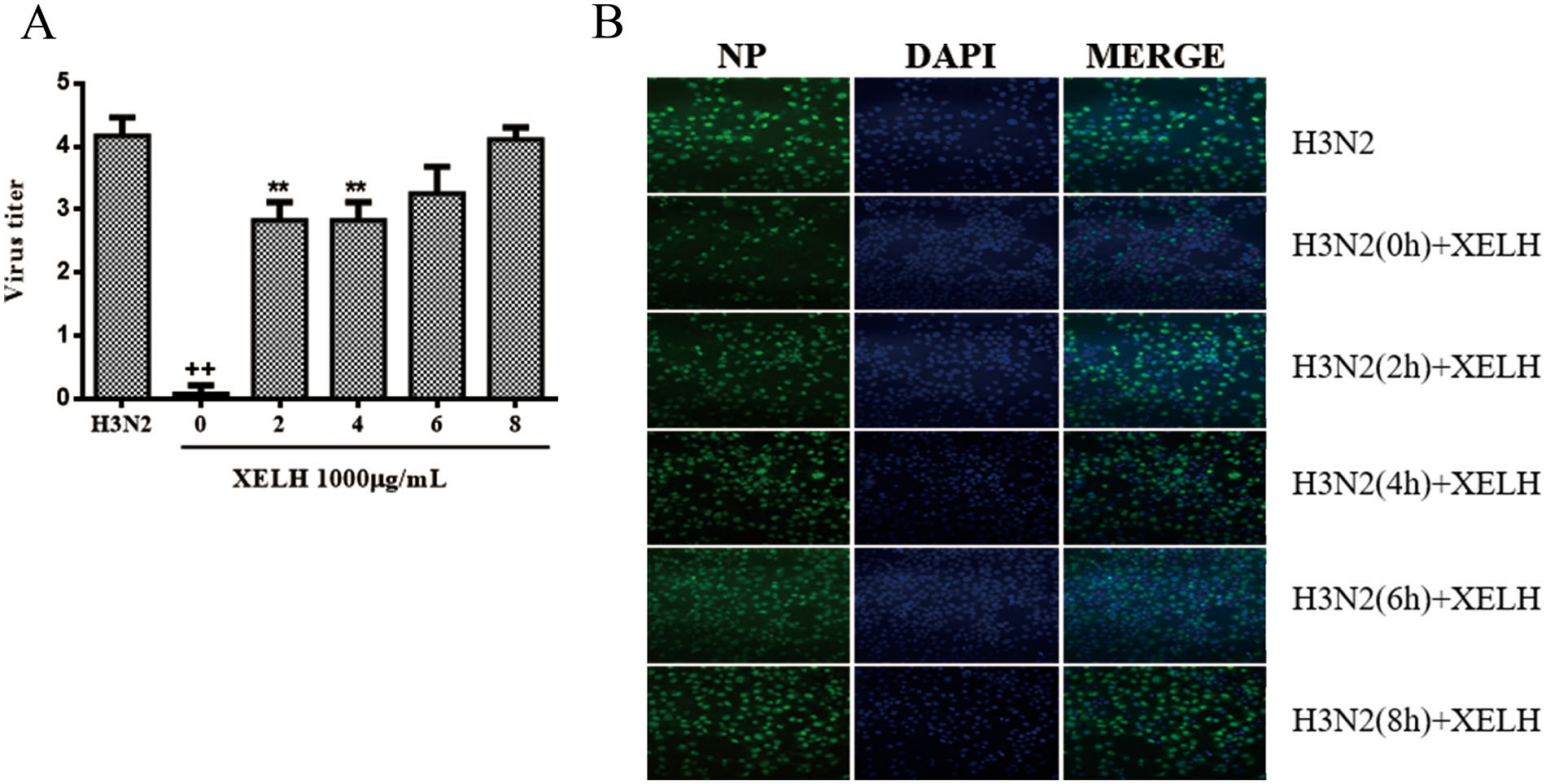
(A) Time-course analysis of effects of XELH (1000 μg/mL) on H3N2. (Data are presented as mean ± SD. ^+^*p* ≤ 0.05, ^++^*p* ≤ 0.01 vs. normal group. *p ≤ 0.05, **p ≤ 0.01 vs. model group.) (B) The effects of XELH on the expression of NP protein of H3N2.

The time-course experiment showed that the virus titre significantly decreased after treatment with XELH (1000 μg/mL) at 0-4 h in the viral replication cycle (Figure 4A). The viral NP decreased in XELH group (treated with 1000 μg/mL XELH) at 0 h compared with the viral infection group (Figure 4B), suggesting that XELH was mainly involved in the early stage of H3N2 virus replication.

### Anti-influenza a virus activity of XELH in vivo

#### XELH reduces body weight loss, HA titre, and lung index in mice infected with H3N2

**Figure 5.**
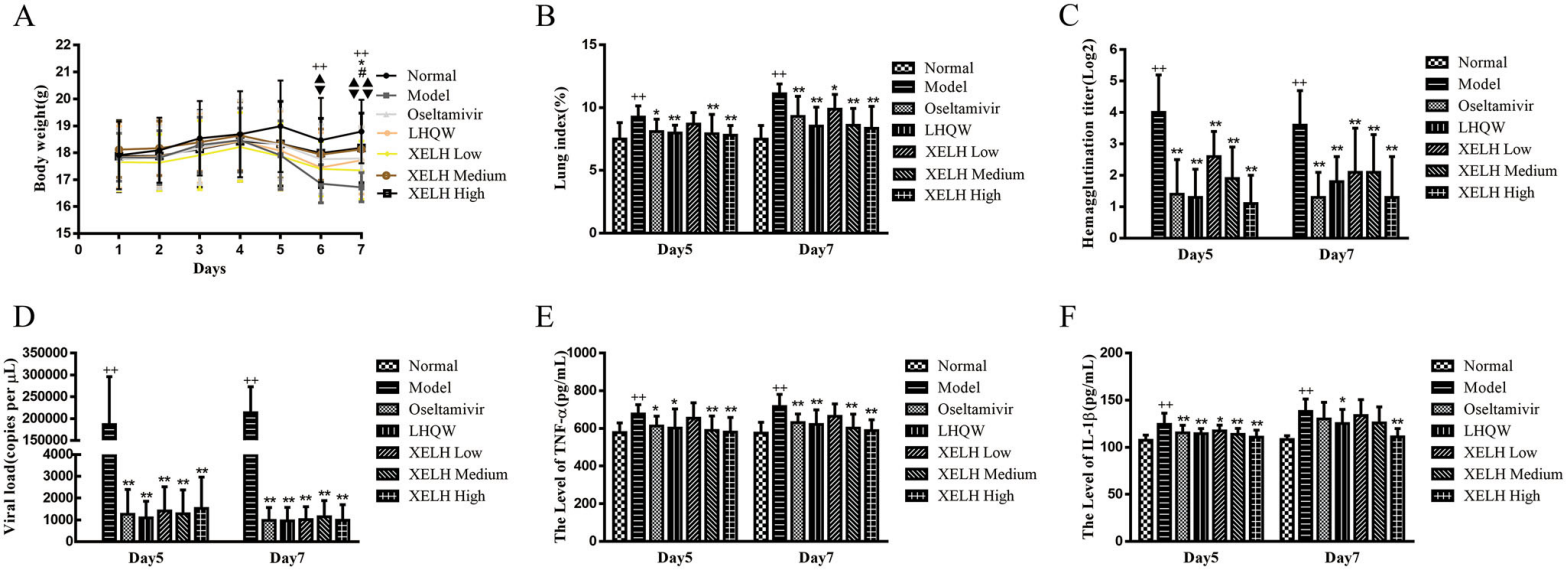
(A) Effects of XELH on bodyweight in mice infected with H3N2. Data are presented as mean ± SD (*n* = 20 in each group). ^+^*p* ≤ 0.05 ^++^*p* ≤ 0.01, Model group vs. Normal group; *p ≤ 0.05, **p ≤ 0.01, LHQW group vs. Model group; ^#^*p* ≤ 0.05, ^##^*p* ≤ 0.01, Oseltamivir group vs. Model group; ▲*p* ≤ 0.05, ▲▲*p* ≤ 0.01, XELH Medium group vs. Model group; ▼*p* ≤ 0.05, ▼▼*p* ≤ 0.01, XELH High group vs. Model group. Effects of XELH treatment on (B) lung index; (C) viral HA titre; (D) H3N2 virus load in lung tissues; (E) TNF-α level in lung tissues; (F) IL-1β level in lung tissues in mice infected with H3N2. Oseltamivir (20 mg/kg), LHQW (0.5 g/kg), XELH Low (2 g/kg), XELH Medium (4 g/kg), and XELH High (8 g/kg). Data are presented as mean ± SD (*n* = 10). ^++^*p* ≤ 0.01 vs. Normal group; *p ≤ 0.05, **p ≤ 0.01 vs. Model group.

H3N2 significantly reduced body weight on D6-D7 (48 and 72 h after the last dose of injection of the virus) in Model group than that in Normal group (*p* ≤ 0.01; Figure 5A). Compared to Model group, the body weight of H3N2-infected mice in Oseltamivir and LHQW groups was significantly increased on D7 (*p* ≤ 0.05), and the body weight of XELH-Medium and XELH-High groups was significantly increased on D6-D7 (*p* ≤ 0.01). The results showed that oral administration of XELH, LHQW, and oseltamivir relieved weight loss in mice infected with H3N2.

On D5 and D7, the HA titre and lung indices between Model and Normal groups were significantly different (*p* ≤ 0.01). The HA titres were significantly decreased in Oseltamivir, LHQW, and all XELH (Low, Medium, and High) groups compared to that in Model group (*p* ≤ 0.01). Furthermore, the lung index also decreased significantly in all treated groups on D5 and D7, except for that in XELH-Low group on D5 (Figure 5B, C).

#### XELH reduces virus load and inflammatory factor expression in the mice infected with H3N2

Figure 5D shows that the virus load was increased on D5 and D7 in Model group compared to that in Normal group (*p* ≤ 0.01). In contrast, it was reduced in Oseltamivir, LHQW, and all XELH groups on D5 and D7 compared to that in Model group (*p* ≤ 0.01).

Compared to Normal group, the expression of TNF-α and IL-1β was increased in Model group on D5 and D7 (*p* ≤ 0.01; Figure 5E, F), indicating that H3N2 infection could promote the release of TNF-α and IL-1β. Compared to Model group, the expression of TNF-α was significantly decreased in the Oseltamivir, LHQW, XELH-Medium, and XELH-High groups on D5 and D7 (*p* ≤ 0.01 or *p* ≤ 0.05). Similarly, the expression of IL-1β was reduced in all treated groups on D5 (*p* ≤ 0.01) and in LHQW and XELH-High groups on D7 (*p* ≤ 0.01 or *p* ≤ 0.05).

#### XELH alleviates the lung tissue damage caused by H3N2 infection

**Figure 6.**
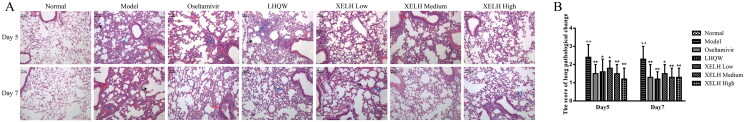
Effects of XELH on lung histopathology in mice infected with H3N2. (A) Effect on lung histopathology after administration. (B) The lung pathological change score on days 5 and 7 (D5 and D7). Black arrows, alveoli; Blue arrows, lung stroma; Red arrows, bronchioles. Oseltamivir (20 mg/kg), LHQW (0.5 g/kg), XELH Low (2 g/kg), XELH Medium (4 g/kg), and XELH High (8 g/kg). Data are presented as mean ± SD (*n* = 10 in each group). ^++^*p* ≤ 0.01 vs. Normal group; *p ≤ 0.05, **p ≤ 0.01 vs. Model group.

As shown in Figure 6, compared to Normal group, the H3N2 infection-induced acute lung tissue damages were prominent on D5. The symptoms included swollen, partially collapsed, and broken alveolar walls; infiltration of inflammatory cells; thickening and some mucosal cells falling off the capillary bronchial wall. The H3N2 infection-induced acute lung tissue damage was improved in Oseltamivir, LHQW, and XELH groups on D5. On D7, part of the alveolar wall collapsed and broken. The pulmonary stroma was thickened with inflammatory cell infiltration in Model group, which was improved in Oseltamivir, LHQW, and all XELH groups. Compared to Model group, the lung pathology score decreased significantly in all treated groups on D5 and D7 (*p* ≤ 0.01 or *p* ≤ 0.05). These results indicate that XELH alleviated H3N2 infection-induced lung tissue damage.

#### XELH improves lymphocytes ratio imbalance in lung tissue

**Figure 7.**
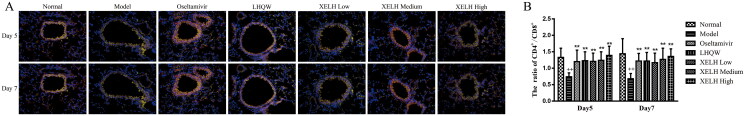
Effect of XELH on the CD4^+^ and CD8^+^ cells in lung tissue of mice infected with H3N2. (A) Effect of XELH on the fluorescence intensity of CD4^+^ and CD8^+^ cells. (B) Effect of XELH on CD4^+^/CD8^+^ ratio in lung tissue. Red fluorescence, CD4^+^ cells; Green fluorescence, CD8^+^ cells; the fluorescence signals were analysed using Image J; fluorescence value represented the number of CD4^+^ and CD8^+^ cells. Oseltamivir (20 mg/kg), LHQW (0.5 g/kg), XELH Low (2 g/kg), XELH Medium (4 g/kg), and XELH High (8 g/kg). Data are presented as mean ± SD (*n* = 6). ^++^*p* ≤ 0.01 vs. Normal group; *p ≤ 0.05, **p ≤ 0.01 vs. Model group.).

Figure 7 shows that the CD4^+^/CD8^+^ ratio in the lung tissues was significantly reduced in the H3N2 infection Model group (*p* < 0.01), suggesting that H3N2 virus infection could cause an imbalance in the CD4^+^/CD8^+^ ratio in the lung tissues of H3N2‑infected mice. The CD4^+^/CD8^+^ ratio in all treated groups increased significantly on D5 and D7 (*p* ≤ 0.01), indicating that XELH improved the imbalance of CD4 ^+^ and CD8^+^ lymphocytes in the lung tissues of infected mice.

### XELH has an anti-respiratory syncytial virus (RSV) infection effect in vivo

#### XELH relieves body weight loss and reduces lung index in RSV-infected mice

**Figure 8.**
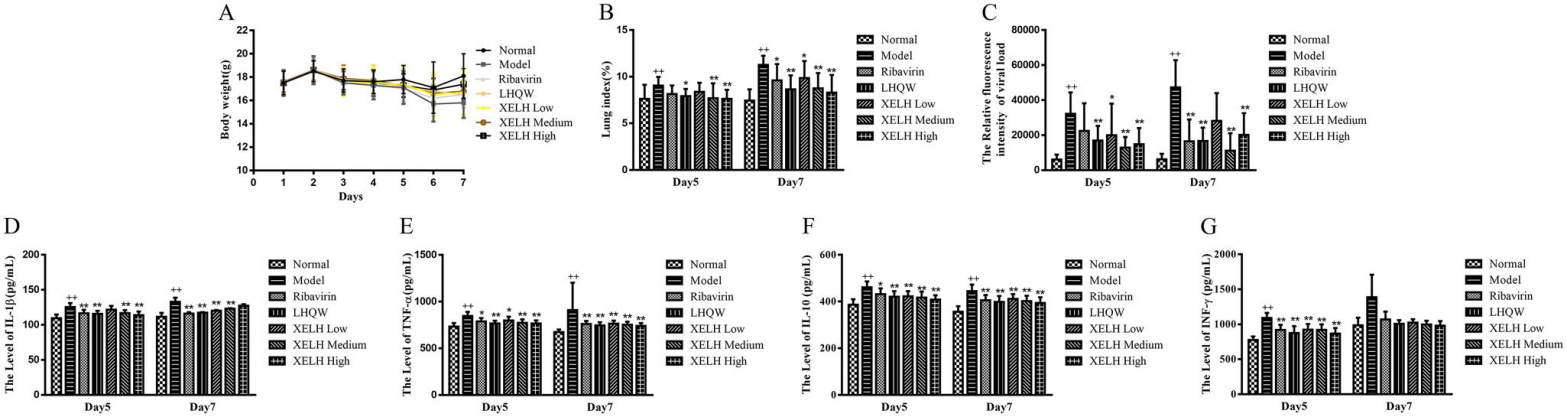
(A) Effects of XELH on body weight in mice infected with respiratory syncytial virus (RSV). Data are presented as mean ± SD (*n* = 20). Effects of XELH on (B) lung index; (C) virus load in lung tissues; (D) IL-1β level in lung tissue; (E) TNF-α level in lung tissues; (F) IL-10 level in lung Tissues; (G) IFN-γ level in lung tissues. Ribavirin (60 mg/kg), LHQW (0.5 g/kg), XELH Low (2 g/kg), XELH Medium (4 g/kg), and XELH High (8 g/kg). Data are presented as mean ± SD (*n* = 10). ^++^*p* ≤ 0.01 vs. Normal group. *p ≤ 0.05, **p ≤ 0.01 vs. Model group.

Figure 8A shows that RSV infection increased the weight loss on D5-D7 (24 and 72 h after the last dose of injection of the virus) in Model group than that in Normal group, but the difference was not statistically significant. Similarly, compared to Model group, the body weight on D5-D7 in LHQW, Ribavirin, and all XELH groups showed an increasing trend (*p* > 0.05).

Compared to Normal group, the lung index was increased in Model group on D5 and D7 (*p* ≤ 0.01; Figure 8B). In contrast, it was significantly reduced in all treated groups on D5 and D7, except for XELH-low group on D5 (*p* ≤ 0.05 or *p* ≤ 0.01).

#### XELH reduces virus load and the expression of inflammatory cytokines in RSV‑infected mice

Compared to Normal group, the relative fluorescence intensity of the virus load was increased in Model group on D5 and D7 (*p* ≤ 0.01; Figure 8C). However, the relative fluorescence intensity of the virus load was reduced on D5 in Ribavirin group (*p* > 0.05), LHQW (*p* ≤ 0.01), and all XELH groups (*p* ≤ 0.01 or *p* ≤ 0.05) compared to that in Model group. On D7, the virus load was reduced in LHQW (*p* ≤ 0.01), Ribavirin (*p* ≤ 0.01), XELH-Medium (*p* ≤ 0.01), and XELH-High groups (*p* ≤ 0.01) compared to that in Model group. Although it showed a decreasing trend in XELH-Low group, the reduction was not significant (*p* > 0.05).

The levels of IL-1β, TNF-α, and IL-10 were significantly increased in Model group on D5 and D7 than those in Normal group (*p* ≤ 0.01) (Figure 8D-F). On the contrary, the level of IL-1β was significantly reduced in all treated groups on D5 and D7 (*p* ≤ 0.01), except for XELH-Low group on D5 (*p* > 0.05) and XELH-High group on D7 (*p* > 0.05) compared to that in Model group (Figure 8D). The TNF-α and IL-10 levels were significantly reduced in Ribavirin, LHQW, and all XELH groups on D5 and D7 (*p* ≤ 0.05 or *p* ≤ 0.01) than that in Model group (Figure 8E, F). Furthermore, the IFN-γ level was significantly increased in Model group (*p* ≤ 0.01). In contrast, it was significantly reduced in Ribavirin, LHQW, and all XELH groups on D5 (*p* ≤ 0.01) compared to that in Model group. On D7, similar trends of changes in IFN-γ level were observed between Model, Normal Ribavirin, LHQW, and all XELH groups; however, the changes were not significantly different (Figure 8G).

#### XELH alleviates the lung tissue damage caused by RSV infection

**Figure 9.**
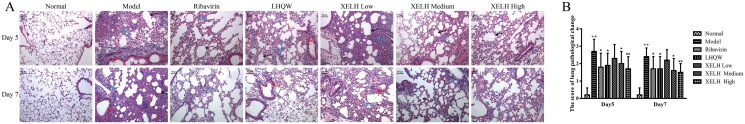
Effects of XELH on lung histopathology of mice infected with RSV. (A) Representative images of lung histopathology after administration. (B) The lung pathological change score on D5 and D7. Black arrows, alveoli; Blue arrows, lung stroma; Red arrows, bronchioles. Ribavirin (60 mg/kg), LHQW (0.5 g/kg), XELH Low (2 g/kg), XELH Medium (4 g/kg), and XELH High (8 g/kg). Data are presented as mean ± SD (*n* = 10). ^++^*p* ≤ 0.01 vs. Normal group. *p ≤ 0.05, **p ≤ 0.01 vs. Model group.

Compared to Normal group, the RSV infection-induced acute lung tissue damage with swollen, partially collapsed, and broken alveolar wall; thickened and some mucosal cells falling off of the capillary bronchial wall; and infiltration of inflammatory cells was more prominent in Model group on D5 (Figure 9). On D7, part of the alveolar wall collapsed and broke, and the pulmonary stroma and bronchial wall were thickened with inflammatory cell infiltration in Model group. These pathological features were improved, and the score of lung pathology decreased significantly in all treated groups on D5 and D7, except for XELH-Low group on D5 compared to those in Model group (*p* ≤ 0.01 or *p* ≤ 0.05). These results indicated that XELH alleviated the RSV infection-induced acute lung tissue damage.

#### XELH improves lymphocytes ratio imbalance

**Figure 10.**
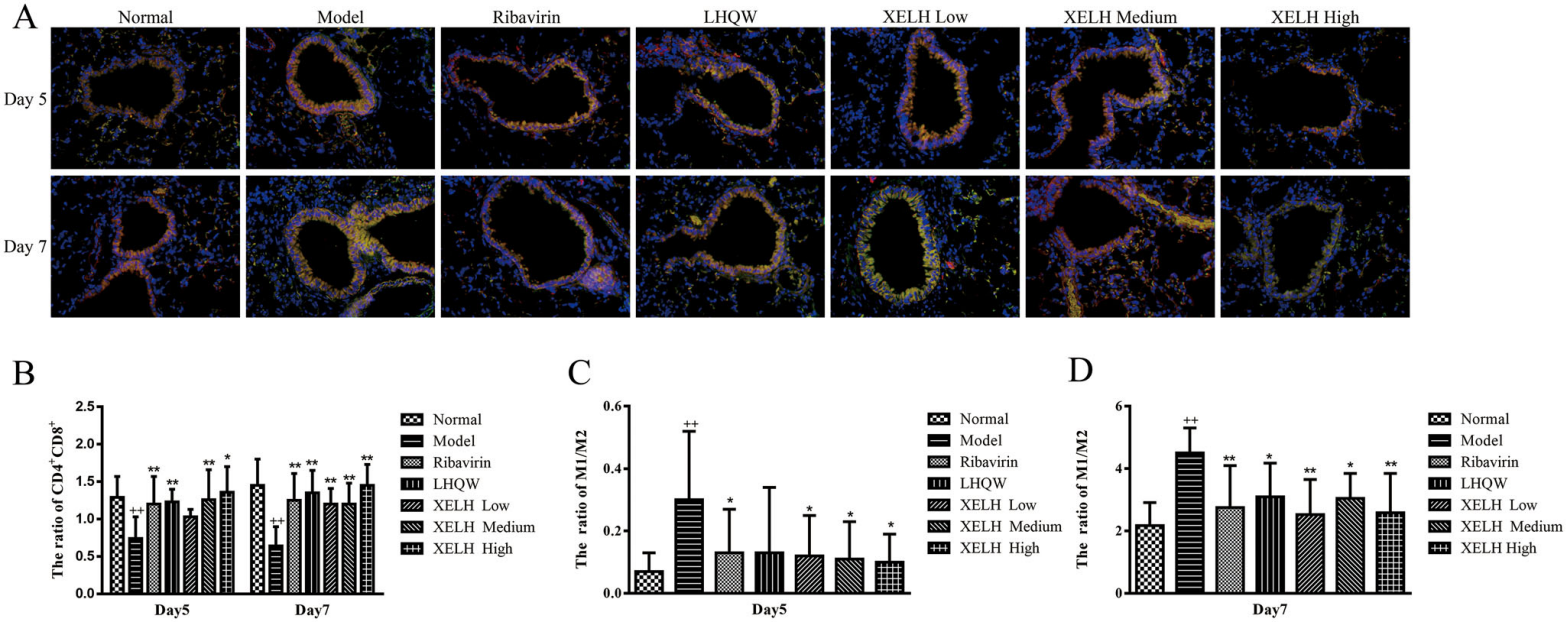
Effect of XELH on the CD4^+^, CD8^+^, M1, and M2 cells in lung tissue of mice infected with RSV. (A) Effect of XELH on the fluorescence intensity of CD4^+^ and CD8^+^ cells; ×400 (B) Effect of XELH on CD4^+^/CD8^+^ immunohistochemical fluorescence intensity ratio in lung tissue. (C, D) Effect of XELH on pulmonary macrophages. Red fluorescence, CD4^+^ cells; Green fluorescence, CD8^+^ cells. The fluorescence signals were analysed using Image J, and the fluorescence value was considered the number of CD4^+^ and CD8^+^. Ribavirin (60 mg/kg), LHQW (0.5 g/kg), XELH Low (2 g/kg), XELH Medium (4 g/kg), and XELH High (8 g/kg). Data are presented as mean ± SD (*n* = 6). ^++^*p* ≤ 0.01 vs. Normal group; *p ≤ 0.05, **p ≤ 0.01 vs. Model group.

Compared to Normal group, the CD4^+^/CD8^+^ ratio in lung tissues was significantly reduced after RSV infection (*p* < 0.01) (Figure 10A, B), suggesting that RSV infection caused an imbalance in the CD4^+^/CD8^+^ ratio in the lung tissues. On D5, the CD4^+^/CD8^+^ ratio in all treated groups was significantly increased except for the XELH-Low group than that in Model group. Furthermore, on D7, the CD4^+^/CD8^+^ ratio in all treated groups was increased significantly (*p* ≤ 0.01). These results indicated that XELH improved the imbalance of CD4^+^ and CD8^+^ lymphocytes in the lung tissue of RSV-infected mice.

#### XELH inhibits the macrophage M1 polarization in lung tissue

As shown in Figure 10C, D, compared to Normal group, the M1/M2 ratio of macrophages in Model group increased significantly after RSV infection (*p* ≤ 0.01) on D5 and D7. Compared to Model group, the M1/M2 ratio in Ribavirin and all XELH groups decreased significantly on D5 (*p* ≤ 0.05); however, the ratio increased nonsignificantly in LHQW (*p* > 0.05). On D7, the ratio of M1/M2 in all treated groups decreased significantly (*p* ≤ 0.05, *p* ≤ 0.01).

### XELH has an anti-inflammatory effect in vivo

#### XELH inhibits the fever induced by injection of LPS in rats

**Figure 11.**
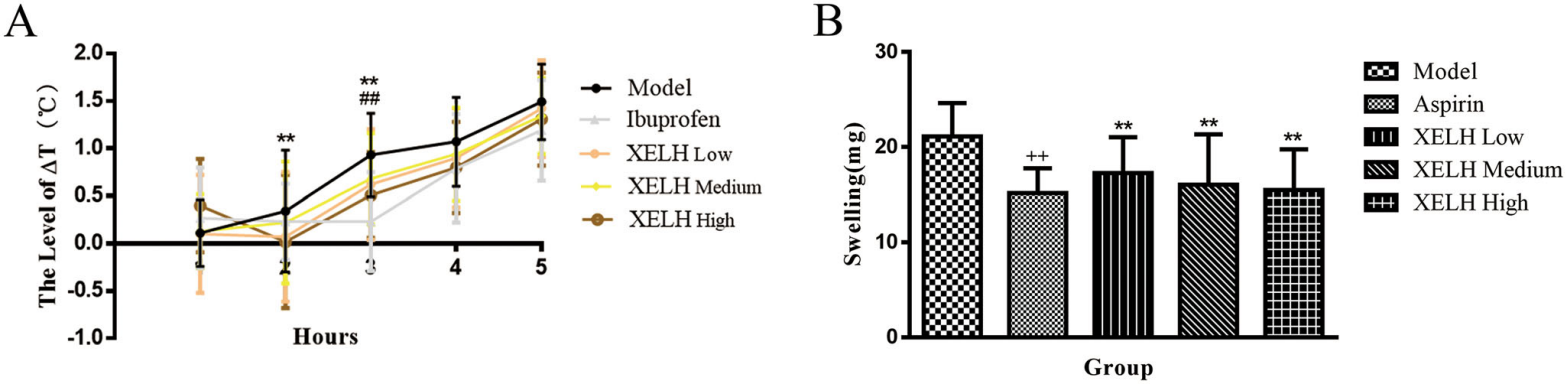
(A) Effect of XELH on LPS-induced fever in rats. Data are presented as mean ± SD (*n* = 18). *p ≤ 0.05, **p ≤ 0.01, Ibuprofen group vs. Model group; ^#^*p* ≤ 0.05, ^##^*p* ≤ 0.01, XELH-High vs. Model group. (B) Effect of XELH on xylenes induced ear swelling in mice. Data are presented as mean ± SD (*n* = 15). ^++^*p* ≤ 0.01 vs. Normal group; *p ≤ 0.05, **p ≤ 0.01 vs. Model group.

As shown in Figure 11A, the temperature of the rats increased 1-5 h after the intraperitoneal injection of LPS in Model group. Compared to Model group, the temperature of rats in Ibuprofen and XELH-High groups decreased significantly at 2 or 3 h after LPS injection (*p* < 0.01).

#### XELH inhibits the ear swelling stimulated by xylene in mice

The degree of swelling was low in treated mice than those in Model mice (*p* < 0.01; Figure 11B).

## Discussion

In this study, we investigated the therapeutic effects of XELH in treating respiratory viral infections. The *in vitro* screening studies indicated that XELH had significant broad-spectrum antiviral effects against several viruses, including influenza A, B, RSV, and coronavirus. XELH inhibited the pro-inflammatory factors in virus-infected host cells. Sequentially, mouse models of acute respiratory virus infections (H3N2 and RSV) were established to confirm antiviral capacities and better understand the underlying mechanisms by which XELH exerts broad-spectrum antiviral activities.

Natural herbal medicine is widely used to prevent and treat viral infectious diseases in China. Many functional components isolated from herbal medicines have been proven to exert antiviral effects *in vitro* and *in vivo* (Li and Peng 2013; Bouvier and Lowen 2014). Given the complicated chemical composition of XELH, we conducted *in vitro* screening assays in the antiviral spectra to identify its SI and safety potential. As shown in Table 3, the SI that indicates the window between cytotoxicity and antiviral activity was calculated by the ratio of TC_50_ to IC_50_: a sample with an SI value ≥ 10 is assumed to have the potential for further investigation. Accordingly, it has been suggested that XELH has great potential as a broad-spectrum antiviral (Indrayanto et al. 2021). Moreover, mounting evidence indicates that the antiviral effects of TCMs used to control respiratory virus infections might be attributed to their immunoregulatory effects rather than selective antiviral effects (Lee et al. 2021).

Infected cells respond in various ways to limit the spread of the virus. For instance, type I interferons (IFN-α/β) are key cytokines produced by epithelial cells and monocytes/macrophages (Högner et al. 2013; Herold et al. 2015). In addition, epithelial cells produce reduced upon activation, normal T cell expressed and secreted (RANTES), MCP-1, and IL-8, while monocytes/macrophages secrete MIP-1α, MIP-1β, RANTES, MCP-1, MCP-3, MIP-3α, and IP-10. Accordingly, several different transcription factor systems are activated to regulate the temporal and spatial expression of genes that produce these chemokines and cytokines (Matsukura et al. 1996; Sprenger et al. 1996; Adachi et al. 1997; Bussfeld et al. 1998). In this study, A549 cells infected with H3N2 showed an increased release of pro-inflammatory cytokines and chemokines, such as MIP-1β, IFN-β, IP-10, IL-6, CCL5, MCP-1, TNF-α, RIG-I, MIG, and CXCL1, wherein XELH suppressed these pro-inflammatory cytokines in a dose-dependent manner. To confirm and validate whether this anti-inflammatory effect is specific to viral infection, *in vitro* LPS-induced activation of macrophages was established. Upon stimulation with LPS, RAW 264.7 cells secreted pro-inflammatory cytokines, such as TNF-α, IL-6, IP-10, MCP-1, and IL-1β, and XELH significantly reduced the expression of pro-inflammatory cytokines. Moreover, XELH exhibited great potential to inhibit the replication of the influenza A virus and downregulate the expression level of influenza virus NP *in vitro*. These results demonstrated that XELH exerted *in vitro* antiviral activities and suggested that its immunomodulatory capacity contributed to the broad-spectrum antiviral activities of XELH.

To further support our *in vitro* findings, we used mouse models of RSV and H3N2 infection to validate the antiviral effects *in vivo*. Generally, pneumonia caused by respiratory virus infection is characterized by weight loss, where the degree of reduction indicates the severity of the viral infection (Sheahan et al. 2020; Wang et al. 2021). In lung tissue, viral replication paralleled indirect antigen titres in lung homogenate supernatants and was reflected by the increased lung indices. Furthermore, immunologic mechanisms contribute to the pathogenesis of viral pneumonia in BALB/c mice. Similar to the infections of RSV and Influenza A virus in humans, IFN-γ has been shown to be the predominant cytokine produced, accompanied by high expression of other cytokines, such as TNF-α and IL-1β in an immunocompromised BALB/c mouse model (Kong et al. 2005). XELH prevented progressive weight loss and decreased lung index, viral load, and IHA antibody titre (Figures 5A-D and 8A-C). Phenotype-based findings confirmed the antiviral potential of XELH against distinct respiratory virus infections. H&E staining results showed that XELH could significantly reduce lung lesions, including the improvement in alveolar structure, inflammatory cell infiltration, and the thickness of the alveolar interstitium, suggesting that XELH could suppress lung tissue remodelling caused by viral inflammation (Figures 6 and 9). In addition to the morphological changes, the expression levels of inflammatory cytokines, including TNF-α, IL-1β, and IL-10, in the infected pulmonary tissues decreased after treatment with XELH. Although the levels of IFN-γ were also reduced by XELH treatment in RSV-infected mice, its expression in H3N2 virus-infected mice was increased over time and decreased when the virus was eliminated. This could be attributed to the heterogeneity in the recovery ability of mice, which could have resulted in instability of the expression level of IFN-γ, especially in Model group. From the data presented, the tendency to inhibit the expression level of INF-γ on D7 was consistent with D5. However, the statistical differences could not be obtained due to the excessive standard deviation (Figures 5E, F and 8D-G).

To gain more insight into the role of XELH in immunoregulation, we performed a T-lymphocyte subtype assay in lung tissue, which revealed the adaptive immune response during viral infection. CD4^+^ and CD8^+^ cells maintain a balance in the body to maintain a normal immune state, which is reflected by the ratio of CD4^+^/CD8^+^ cells (Kruijsen et al. 2010). However, during an interplay between respiratory viruses and the immune system, dysregulation in the T cell subset alters the CD4^+^ and CD8^+^ T lymphocyte population, leading to an imbalance in the CD4^+^/CD8^+^ ratio and aberrant cytokine response (Kruijsen et al. 2010; Chaple et al. 2021). In this study, the balance of the CD4^+^/CD8^+^ ratio in lung tissue was disturbed by RSV and H3N2 influenza A virus infection, and XELH reversed the imbalance in CD4^+^/CD8^+^ ratio in a dose-dependent manner (Figures 7 and 10A-C).

RSV infection is associated with a mix of Th1 and Th2 cytokine storms and is the most important cause of lower respiratory tract infections in infants (Boukhvalova et al. 2006; Wang et al. 2014). During the initial stages of RSV infection, RSV induces the expression of various antiviral genes that drive the development of macrophages towards an antiviral and pro-inflammatory M1 phenotype (Griffiths et al. 2017; Glaser et al. 2019). We found that XELH inhibited macrophage M1 polarization in a dose-dependent manner in the lung tissue (Figure 10D), which indicated that XELH could also coordinate innate immunity to suppress inflammatory responses in mice infected with RSV.

Although typical examples of respiratory infection in mice confirmed the antiviral screening experiments, the protective effects of XELH in respiratory virus infections may not be exclusively attributed to immunoregulation. The complexity of the components of TCM determines its multiple therapeutic targets. Therefore, we have planned to study the active pharmacotherapeutic components based on pharmacokinetic studies and the underlying molecular targets by which XELH serves as a broad-spectrum antiviral medicine.

## Conclusion

Our results demonstrated that XELH exerts a broad-spectrum antiviral effect on respiratory virus infection *in vitro* and *in vivo*. Due to the advantage of multi-ingredients, XELH not only displays the activity in directly inhibiting virus infection but also has an antiviral effect on host immunoregulation. It is worth noting that XELH, as a paediatric medication has a definite antiviral effect on RSV in preclinical studies, which suggests the clinical trial on RSV infection. Given no specific treatment for RSV infection, XELH may be a promising option to address the shortage of therapeutic agents in clinical settings.

## Data Availability

The datasets used in the current study are available from the corresponding author on reasonable request.

## Geolocation information

Shijiazhuang Yiling Pharmaceutical Co., Ltd, No.238, The South of Tianshan Street, Shijiazhuang 050035, Hebei, China.
